# Correction: Identifying the key regulators that promote cell-cycle activity in the hearts of early neonatal pigs after myocardial injury

**DOI:** 10.1371/journal.pone.0284835

**Published:** 2023-04-18

**Authors:** The PLOS ONE Staff

There are errors in the Funding statement. The correct Funding statement is as follows: This work was supported in part, by the National Institutes of Health NHLBI R01 grants HL 95077, HL 149137, and U01 HL134764. No additional external funding was received for this study.

There are also errors in the Competing Interests statement. The correct Competing Interests statement is as follows: The authors have declared that no competing interests exist.

The publisher apologizes for these errors.
