# Supplementary material for: Identifying the key regulators that promote cell-cycle activity in the hearts of early neonatal pigs after myocardial injury
Source: PLoS One. 2020 Jul 30;15(7):e0232963. doi: 10.1371/journal.pone.0232963 (PMC7392272; doi:10.1371/journal.pone.0232963)
Supplement: S1 Raw images — (PDF) [file pone.0232963.s001.pdf]

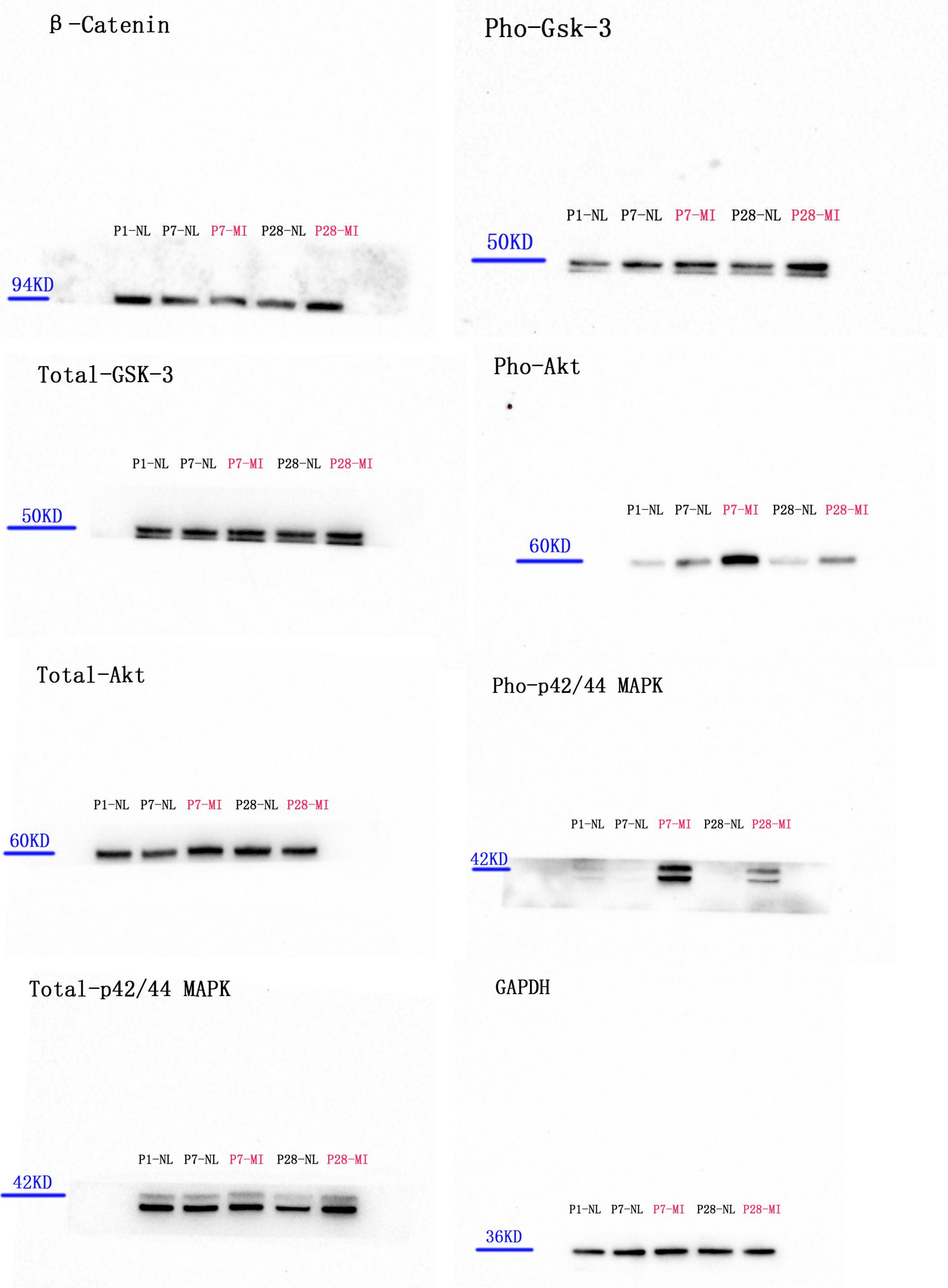

The PVGF membrane was incubated with HRP-conjugated secondary antibodies for half an hour and rinsed with wash buffer, then enhanced chemiluminescence (**ECL**) was added and images were captured in a Biorad Gel Doc System.
