# Supplementary material for: Identifying the key regulators that promote cell-cycle activity in the hearts of early neonatal pigs after myocardial injury
Source: PLoS One. 2020 Jul 30;15(7):e0232963. doi: 10.1371/journal.pone.0232963 (PMC7392272; doi:10.1371/journal.pone.0232963)
Supplement: Attachment — Submitted filename: 3-11-Response to WB-2020 PONE.docx [file pone.0232963.s004.docx]

March 11, 2020

**Professor Guo-Chang Fan, PhD
PLOS ONE, Academic Editor**

**RE: Manuscript # PONE-D-19-33077
“Identifying the Key Regulators that Promote Cell-cycle Activity in the Hearts of Early Neonatal Pigs after Myocardial Injury”**

Dear Dr. Fan,

We appreciate the editor careful review and suggested clarifications in your email dated 3/3/2020 7:33AM

A point-by-point response to their comments are provided following this cover letter. Manuscript has been improved significantly according the reviewer’s suggestions. Enclosed please find our manuscript entitled " Identifying the Key Regulators that Promote Cell-cycle Activity in the Hearts of Early Neonatal Pigs after Myocardial Injury” by Drs. Eric Y Zhang et al. This is a research paper. We investigated whether They found that five signaling pathways (MAPK, Hippo, cAMP, JAK/STAT, and Ras) were upregulated following neonatal cardiac injury, and suggests that these pathways may contribute to regenerative capacity of neonatal porcine hearts. We would like this manuscript to be considered for publication in PLoS One. All the authors have read and approved submission of the manuscript. The manuscript has not been published and is not being considered for publication elsewhere in whole or part in any language.

Thank you for your considerations.

Sincerely,

Jianyi “Jay” Zhang, M.D., Ph.D.

Professor of Medicine, of Engineering,

School of Medicine, School of Engineering

***Response to Reviewers***

***(March 11, 2020)***

***Manuscript # PONE-D-19-33077
“Identifying the Key Regulators that Promote Cell-cycle Activity in the Hearts of Early Neonatal Pigs after Myocardial Injury”***

***By Eric Y Zhang et al***

We the editorial office careful and constructive critique of **Figure 6**. A point-by-point response to their comments are provided as follows.

1. **None of the images provided for b-catenin or Akt appear to match the images shown in the figure. Can you please comment on this and provide the underlying data for the images reported in these figure panels?**

**Response:** Thank you for your question. The experiment of WB has been redone, and the **Figure 6** of the revised manuscript has increased clarity. Specifically, the raw data of b-catenin or Akt is in the previous folder, but their background and contrast have been adjusted by the software before being placed in the figure, so it may look a little different. We have reorganized the data and all the images shown in the figure are now from folder 1.

***Figure 6. Western blotting. LAD ligation at P1 activates key cell proliferation signaling pathways and downstream components.*** *The expression of β-catenin, phosphorylated (Pho-) GSK 3α/β, total GSK 3α/β, Pho-Akt, total Akt, Pho-p42/44 MAPK, and total p42/44 MAPK was evaluated via Western blot. GAPDH levels were also evaluated to confirm equal loading.* ***(A)*** *Representative Western blotting of hearts from each time point/group;* ***(B)*** *Compiled Western blotting data, n=3 hearts each bar. T-test ( 2 tails) with Bonferroni correction. *, p<0.05 between P28-MI vs P28-NL; #, p < 0.05 vs P7-MI; †, p<0.05 vs P7-NL.* *Total protein blots were the same original blots stripped and reprobed.*

2. **Please provide lane loading information for all raw blot data files, either by adding labels directly in the image files or by providing a supporting file that lists the lane loading information for all blot files.  (Several images provided include more lanes than are shown in the figure.)**

**Response:** Thank you for your question. Loading information for all raw blot data files are now the same: P1-normal heart, P7-normal heart, P7- myocardial infarction heart (Received LAD ligation at P1), P28-normal heart, P28- myocardial infarction heart (Received LAD ligation at P1). The previous WB bands included some preliminary data. In some groups, we placed more than one sample, so it looks not clear enough.

**3.	The Methods section says, “Phosphorylated proteins were detected first; then, the blots were treated with stripping buffer (Fisher scientific, PI21059) before total protein expression was evaluated..” However, the corresponding images for phosphorylated Gsk3 do not appear to align with the blot images provided for total Gsk3 (e.g. comparing p-Gsk-3beta2.tif vs,. total-GSK3-2.tif). Similarly, the corresponding images for p-Akt and Akt do not appear to correspond to the same original blots, and some p-ERK and ERK images appear to be derived from different blots. Please comment on this and explain how the control blot experiments were performed, for example were the same original blots stripped and reprobed or did you obtain control and experimental data using parallel blots generated from the same protein samples? For data obtained by stripping and reprobing the same blot please ensure that the relationship between the raw image files is evident based on file labels and/or annotation in a supporting information file.**

**Response:** Thank you for raising this question. At the beginning of the experiment, we tried different antibody concentrations and different pig samples, and finally determined the time point we need to set. So the initial data was not strictly arranged according to the grouping in the figure, that is why some phosphorylated proteins do not align with the total proteins. Based on the grouping in figure, we have redone the WB experiment and shown them in the new folders. The original images reported in the figure are all in folder 1. All the control blot experiment were performed as we described in the method, they were the same original blots stripped and reprobed.

**4.	In reviewing the raw blots we noticed that the figure includes blots labelled in your Supporting Information folder with 1, 2, and 3 at the end of the file names. Please clarify whether the panels in the figure all show data from the same internal, matched experiment. At minimum, all data in the figure should have been obtained using the same protein samples, and the figure legend should indicate whether the data were obtained using the same blot (cut into strips, stripped/reprobed, etc.) or whether parallel blots were run with the same protein samples.**

**Response:** Thank you for raising this question. The panels in the figure all show data from the same internal, matched experiment. All data in the figure were obtained using the same protein samples, and all the control blots were the same original blots stripped and reprobed. We have indicated this in the figure legend.

**5. If you have not already, provide the unprocessed individual-level quantitative data underlying the graphs in Figure 6B in supporting information.**

**Response:** We have provide the unprocessed individual-level quantitative data underlying the graphs in Figure 6B in supporting information.

**6. Please review our guidelines for raw blot and gel data at** [**https://journals.plos.org/plosone/s/figures#loc-original-images-for-blots-and-gels**](https://journals.plos.org/plosone/s/figures#loc-original-images-for-blots-and-gels) **and ensure that your data files abide by all of the journal’s requirements for these data files. If you have any questions, please email me**

**Response:** Thank you for your reminders, we have ensured that our data abided by all of the journal’s requirements. We have labeled each raw blot image to clearly annotate the loading order, identity of experimental samples, method used to capture the image. Molecular weight markers were all indicated on the raw image.
